# Assessment of the Status of Rooftop Garden, Its Diversity, and Determinants of Urban Green Roofs in Nepal

**DOI:** 10.1155/2022/6744042

**Published:** 2022-06-06

**Authors:** Sara Rawal, Sandesh Thapa

**Affiliations:** Gokuleshwor Agriculture and Animal Science College, Tribhuvan University, Baitadi, Nepal

## Abstract

In recent days, the practice of adopting rooftop garden can be seen in urban areas of developing countries, but a successful adoption of well-equipped green roofs is still lacking and is limited to open farms. To fulfill the gaps in urban agriculture in determining diversity status and socioeconomic factors affecting the adoption of RTG, this study was conducted. The survey was conducted from February 3 to April 6, 2021, where a total of 116 respondents were selected randomly from Morang and Sunsari districts. The rooftop adopters had 30.5% and 33.2% of the roofs under farming in Morang and Sunsari, respectively, having the size of the roof of rooftop adopters significantly larger than nonadopters. A binary logit model was used to determine the factor affecting the adoption of RTG where age, gender, schooling year, training, and farming experience have a significant effect on the adoption of RTG. Locally available material was given preference under farming and nutritionally important 50 species were reported with tests of the daily food requirement of the respondents. The diversity indices suggest that ornamental plant diversity is more followed by vegetables. Though, adopters are continuing the garden but have reported that lack of proper policy and ineffective management makes it difficult to protect the life of roof. Concludingly, respondents and other willing people must be provided with training, financial support, and proper extension services as lack of training and extension services are the major problems reported in the study area. Proper policy of rooftop garden is lacking in study area though it is under study in Kathmandu; thus, policy makers and research institution should focus on promoting the rooftop in study area and provide more reliable package for roof protection and garden continuation.

## 1. Introduction

Human population is increasing on the Earth at an alarming rate and is also causing social-ecological and environmental pressures. A lot of efforts have been done to ensure the sustainability of the Earth and create ecological balance, still high population density in urban areas in search of quality, service, facilities, jobs, and better opportunities causes environmental pressures in urban areas due to which its environmental attributes are degrading and causing ecological pressures. The major consequence faced is a decrease in food production areas as they are covered by skyscrapers and this is a major issue in developing areas because of unplanned and random urbanization. With regards to this in recent days' concrete roofs of urban areas are being transformed into productive green roofs in developed countries and their advancement is also high as both protected and unprotected roofs are in practice. However, in developing countries, its adoption is likely to be increasing but not all are adopting. Advancement in green roofs is not observed in developing south Asian countries like Nepal [1].

The use of green roofs is dominantly used for fresh vegetable production at a minimal space with a minimal cost to ensure food security in urban areas; however, in developing countries, rooftop farms are more clearly viewed as gardens, and the diversity of flowers is much higher [2, 3]. For developing countries like Nepal, rooftop gardens in urban areas at a noncommercial scale also help to ensure food security and aid in ecosystem services from the local level. The use of roof tops in changing environment is much more beneficial as microclimate modification is the ease and can also be used for off-season purpose. Rooftop farms provide a variety of ecosystem services and for its adoption and continuation, the use of locally available materials is also efficient in providing better production in urban spaces [4].

In Nepal, currently, a limited number of households are adopting rooftop gardening with the support of NGOs, Municipalities, and the Ministry of Agriculture Development (MOAD). Kathmandu Metropolitan Council has decided to provide financial support to 500 households to increase the rooftop gardening program to encourage local food production and municipal waste management sustainably.

For the adoption of rooftop gardens and proper policy level decision-making, social acceptance is of utmost importance as social acceptance will determine the development of urban agriculture and the specific knowledge of citizens' perceptions is required to set the basis for policy-making and planning [5]. Though the program focused on expanding the rooftop garden in Kathmandu, the average area covered by a rooftop garden, its adoption, and the introduction to its importance have not been highlighted in other parts of the country; thus this survey was conducted in one of the fastest-growing cities of Nepal (Biratnagar and Itahari). In the selected area, adoption vs. observation of rooftop gardens can be clearly observed and previously no effort has been made at the societal level to study the determinants of RTG using a binary logit model. Here, adopters were found to continue and even expand their garden; however, the nonpractitioners attitude was not considered so that the influencing factor for adoption by nonpractitioners can be analyzed.

This survey was conducted to highlight the status of rooftop gardening and its adoption. Lack of technical knowledge, availability of true-to-type plant varieties, lack of support and coordination from the government sector, and lack of free time are some difficulties faced by people. Still, more research is going on the implementation and effective performance of green roofs in different parts of the country, but study regarding the factor affecting the adoption of RTG is still not considered. Moreover, research surveys and research studies in the urban setting of a developing country are still lacking in focus on the strength of socioeconomic characteristics in the adoption or rejection of RTG. Thus, this study aims to fulfill the gap in the adoption of RTG in urban settings and assess the diversity status of plant in roof and constraints faced by the adopters vs. the perception of nonadopters.

## 2. Literature Review

In most countries, arable land is already in use leaving meager prospects for its expansion. As per the United Nations, by the year 2025, two-thirds of the world's population would live in water-stressed regions due to climate change. Presently, 70% of the world's fresh water is used for traditional agriculture, so the water crisis would obviously lead to a food crisis. Rooftop or vertical farming makes lesser use of water for crop production. These types of farming are therefore the possible solution to address the impacts of climate change and population growth. The promotion of rooftop of vertical farming should get due consideration in all urban and periurban areas to address the impact of climate change and population growth. Rooftop farms are more economical in construction as compared to vertical farms though vertical farms can be established on roofs with high space utilization and efficiency.

For developing countries like Nepal, urban agriculture plays an important role in terms of both production (crop and vegetable production) and environmental benefits (carbon sequestration). Various studies on roofs have been conducted by several authors in developed countries and still, yield potential and use efficiency of roofs in a developing country is neglected and is getting in practice since the past few years.

### 2.1. Benefits of Rooftop Garden

Rooftop garden are well known for their multiattributes, namely, source of healthy food, fresh air circulation, carbon sequestration, aesthetic purpose, heat reduction in buildings [6]. Pollination systems, urban greenery, biodiversity conservation, and promotion are the underlying beauty and impacts continuation of the rooftop garden [7–10]. Rooftop garden not only enhances the present condition biodiversity but also attracts various insects, birds, and other fauna to the urban green roofs. Soil microbial diversity is one of the important aspects of the agriculture ecosystem, hereby, reported of being promoted in the rooftop garden. New plant species can be reported in RTG because of volunteer crops which are mainly weed species, and sometime crop plants like *Amaranthus* can also be observed. Moreover, inferences from rooftop farming systems and some suitable crop species reported by several authors in rooftop gardens are as presented in Table 1.

## 3. Methodology

### 3.1. Study Area

**Figure 1:**
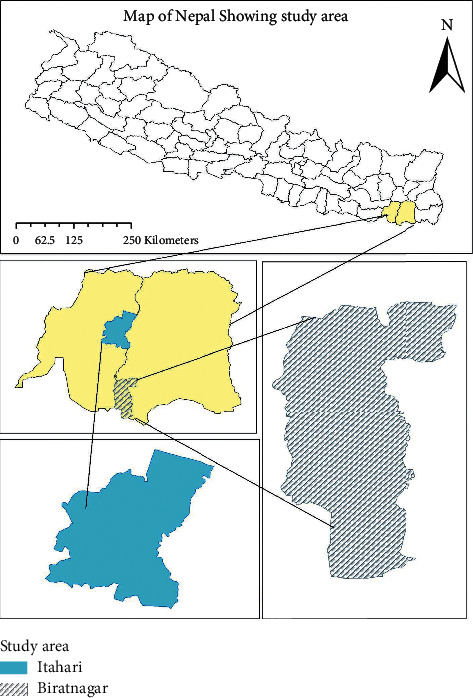
Map of Nepal showing study area (ArcGIS 10.7.1).

The selected study area lies in province number 1 of Nepal, i.e., Morang and Sunsari districts (Figure 1). Biratnagar was selected for study from Morang district which is the capital of province 1 and Itahari was selected from Sunsari. Both study areas have dense populations and visual evidence of practicing rooftop farming was also spotted. Both study areas lie in the Terai region, i.e., they share common agroclimatic features. Itahari is 16 km north of Biratnagar. The average annual temperature of the study area is 30.2°C with annual precipitation of 1891.8 mm [16, 17]. The area and population of Biratnagar are higher than that of Itahari. Biratnagar has a total area of 174 km^2^ and a population of over 24000 whereas the area of Itahari is 93.78 km^2^ and has a population of 140517 [18].

### 3.2. Research Question, Sample Size, and Sampling Technique

A semistructured questionnaire was used for the survey where the questionnaire was divided into three sections and a key informant interview was conducted among the randomly selected respondents. The sample size of the respondents was 58 in each district where respondents were selected randomly without replacement. Simple random sampling is one of the most used and convenient methods of sampling as it reduces biasness among respondents, which has a direct effect on the outcome of the results [19].

The research question was constructed using past literature [4, 6, 20] and past field surveys [1, 20], and constraints were added from pilot testing in the study area based on problems faced by respondents. 10 respondents from each area were interviewed for the piloting of the questionnaire but were not included in the final survey. The survey duration lasted for 2 months and 3 days from February 3 to April 6, 2021. After the completion of the survey, 2 focus group discussions were conducted in each district following health protocols, and the number of respondents who participated were 25 in Morang and 34 in Sunsari where most of them were rooftop nonpractitioners in both study areas.

#### 3.2.1. Institutional Review Board Statement

Before conducting the survey, the objective of the research, its motive, and the targeted audience was discussed at Gokuleshwor Agriculture and Animal Science College, in the Department of Research and Extension Services under the institutional review board. The board approved the proposal and stated that the study was conducted according to the guidelines of the Declaration of Helsinki. The study has been conducted according to established ethical guidelines and informed consent has been obtained from the participants.

#### 3.2.2. Primary Information

Primary information includes the socioeconomic variables used in the survey. In any social science survey, collection of primary information is of utmost importance as it provides a basis for understanding how the society is built up and what sort of aspects to be improved to influence technological innovation. Data collection includes gender, age, schooling year, occupation, farming experience, family size, and economically active population.

#### 3.2.3. Information Related to Green Roofs

This section is focused on the rooftop size and the size of the garden. This section was divided into two different sections for adopters and nonadopters. For adopters and nonadopters, benefits of RTG, its impact, satisfaction, and production efficiency were discussed, along with the prevailing constraints if identified. Both were interviewed for their willingness to take part in training and workshops conducted about rooftop gardens. Adopters were separately interviewed about the total roof area under farming and their response to increasing size or not. Similarly, regarding planting materials, respondents were interviewed and detailed information was collected about planting materials and which were used the most. The adopters were interviewed about the sustainability of rooftop gardens and their role in food self-sufficiency. Most of the information was modified from more than one source including research papers, personal experience, and institutional suggestions.

### 3.3. Diversity Assessment

Diversity is all about how many crops of which species have been reported in the study area and later converted into diversity indices. The total number of each species of the plant reported from the study area was recorded and a diversity assessment was conducted. Plant frequency counts on the rooftop were done on a population basis rather than quadrant and they can mislead to diversity information. The number of species spotted in one quadrant may or may not be spotted in the next quadrant. Thus, rather than the quadrant method, the population method was used and is found to be more effective as the total existing diversity of RTG of study area was assessed present in the study period. The diversity assessment in the study area was analyzed using R Studio V. 4.0.1.

### 3.4. Conceptual Framework and Binary Logit Model

Globally, urban rooftop farming is gaining popularity [10] and is attributed to multiple benefits due to which researchers and policymakers focus on sustainable cities which are directed toward the urban green roof. The adoption of rooftop gardens with various variations like open-air, greenhouse, hydroponics systems, and aeroponics system is in practice in developed countries. However, in a developing country like Nepal, simple open-air rooftop garden can be seen and still many people have not adopted a rooftop garden. Thus, this study is designed with two motives one to address the status of rooftop gardens, and the next is to record the response of nonadopters regarding rooftop gardens.

**Figure 2:**
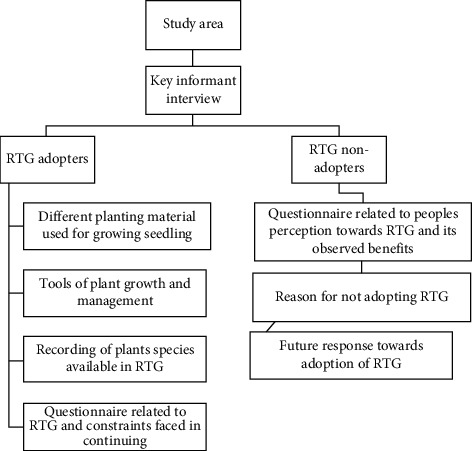
Method of interview and some key questions based on rooftop practitioners in the study area.

**Figure 3:**
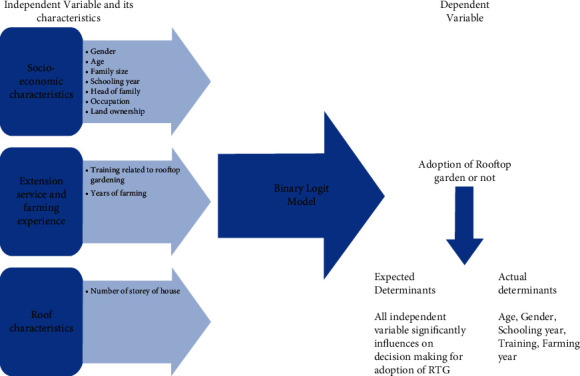
Conceptual framework of using binary logit model and variables used.

Socioeconomic features play a key role in society for adoption options and continuation of the so-called adopted technology. Thus, this study was designed using a binary logit model to determine the factor affecting the adoption of RTG. For determining the adoption of the rooftop garden, we interviewed both practitioners and nonpractitioners and concluded the result. The details of the working methodology and framework have been presented in Figures 2 and 3. The method of interviewing and question at a glance has been presented in Figure 2. The role of the socioeconomic characteristics in the adoption of RTG and willingness to pay for RTG products has been studied by [5], but the degree of adoption and which factor affects how much have not been studied. Also, in developing countries like Nepal adoption is highly influenced by socioeconomic characteristics which have been explained by [21–25].

The binary response model is a regression model in which the dependent variable Y is a binary random variable that takes on only the values zero and one. In predicting the various socioeconomic factors affecting or influencing the adoption of new or improved technologies, the use of the binary logit model is most popular [26]. If the dependent variable has two outcomes, one of them is coded as “one” and the other as “zero” [27].

In this model, *Y* is the adoption of RTG or not and *X* is the various factor affecting the adoption of RTG (Table 2). The likelihood (ratio) of the farmers for the adoption of RTG is determined by the odds ratio, i.e., the ratio of probability *Y* = 1 to *Y* ≠  1 (equation (1)). However, the binary logit regression model is determined by the natural log of odds. Furthermore, the logit regression model with respect to intercept, coefficients, and dependent and independent is shown as follows:

(1) $$\begin{array}{c} \text{Odds}\left(Y\right)=\frac{p\left(y=1\right)}{\left(1-P\right)\left(Y\neq 1\right)}, \end{array}$$

(2) $$\begin{array}{c} \ln\text{Odds}\left(Y\right)=\ln\left[\frac{p\left(y=1\right)}{\left(1-P\right)\left(Y\neq 1\right)}\right]=\log\text{ it}\left(Y\right). \end{array}$$

The final equation of the logit model can be written as [26, 28, 29]:

(3) $$\begin{array}{c} Y=\log\text{ it}\left(Y\right)=\ln\left[\frac{P}{\left(1-P\right)}\right]=a+\beta 1X1+\beta 2X2+\beta 3X3+\beta 4X4+\cdots +\beta 10X10, \end{array}$$

where *P* is the probability of adoption of RTG and 1-P is the probability of not adopting RTG.

### 3.5. Diversity Indices

The four diversity indices used to analyze the results have been analyzed using R-studio v 4.0.1. In accessing the diversity of species in the study area, these four are important and must-use tools to analyze the diversity [30]. The details of the diversity indices have been presented in Table 3. The main reason for using the diversity indices is to access the abundance of the species with respective classes (fruit, vegetable, etc.) so that it helps in understanding the diversity of the study area and species richness.

## 4. Result and Discussion

### 4.1. Socioeconomic Characteristics

Socioeconomic characteristics are one of the important information to be recorded in the surveyed study area as it plays a significant role in technology transfer, adoption, awareness, and understanding of the need of respondents of the study site. In the surveyed area, Morang and Sunsari, the highest number of male respondents were encountered during the survey and their mean age was 38.47 and 42.14 in Morang and Sunsari, respectively. In Morang, Brahmins (25) were found to be dominant in population followed by Chhetri's (15), Madhesis (11), and Janajati (2) whereas in Sunsari, Janajati (24) were reported to be in higher frequency followed by Chhetri (17), Brahmin (13), Madhesis (7), and Dalits (2).

**Figure 4:**
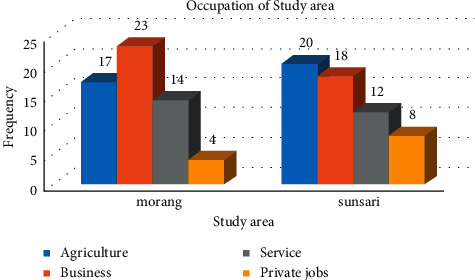
Occupational status of study area.

The average family size was almost the same in both districts, in the range of 5, and the average size of economically active members was 2.12 and 2.14 in Morang and Sunsari and is almost the same. The head of family and decision-making was dominated by a male in both study areas and was found in higher frequency in Sunsari than that in Morang. The average schooling year of the respondents in the study area was 11 years and 89.65% of respondents in Morang and 82.75% of respondents in Sunsari were literate; i.e., literacy rate within the sample frame was significantly higher than the national average. The details of the socioeconomic characteristics have been presented in Table 4 and occupational status has been shown in Figure 4.

### 4.2. Status of Rooftop Garden

**Figure 5:**
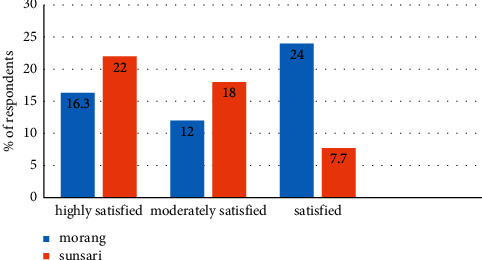
Satisfaction of rooftop adopters in its adoption.

In the study, the area under the rooftop garden was documented from the rooftop adopters and probable adopters (nonadopters), the amount of roof you would like to create a rooftop garden on; this means that the respondents are willing to establish it in the future but have not processed it yet. The study reported that the size of the roof of rooftop garden adopters was significantly higher than that of nonadopters (Table 5). However, the area under the rooftop garden and that to be covered in the future by nonadopters were statistically similar, i.e., nonadopters willingness to adopt the rooftop garden covers a similar area of roof to that of adopters. Also, the status of the rooftop gardens was further compared with the study area, and the size of roof was statistically similar and the area of the roof under farming was significantly different where green roofs were denser in Sunsari as compared to Morang (Table 6). In the study area, no hydroponics and aeroponics systems and cultivation under greenhouses were recorded. All the rooftop practitioners were found to adopt nonprotected farming practices. The above-mentioned practices that were not adopted in the study site were protected systems, having high and more sustained productivity as compared to the nonprotected systems [5]. Regarding the people's perception of the rooftop gardens, frequency of respondents being highly satisfied in Sunsari and satisfied in Morang was maximum. Most of the respondents of Sunsari were highly and moderately satisfied whereas there was not much variation in the satisfaction in the Morang district (Figure 5).

### 4.3. Benefits of Rooftop Garden

**Figure 6:**
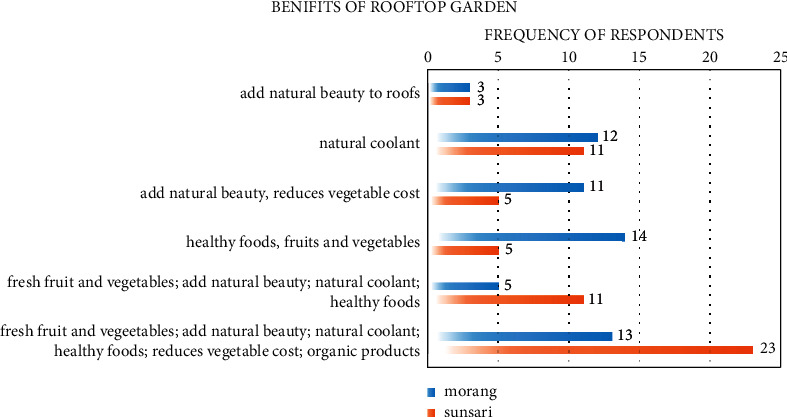
Benefits of rooftop gardens reported in study area.

Urban agriculture like rooftop gardens cultivation, production, and creation in cities provides multiple benefits like self-sufficiency, recreation, and social inclusion. In urban ecosystems, rooftop garden is one of the emerging concepts and was found to have multiattributes. Rowe [35] reported that benefits of rooftop garden considering its limitations before installing green roofs were reduced budget estimation, and weight estimation of roofs should be the primary concern. Green roofs are growing and have a demanding need in urban areas, and they require to be installed for sustained food supply with a reduction in ground water and soil contamination with heavy metals and a growth of healthy food. Roof underfarming serves in a variety of ways as suggested by literature and a similar approach was used to identify the benefits by rooftop adopters. One of the major benefits of rooftop farming is that it does not compete with other land uses and provides sustainability in production and ecosystem [10]. In Morang, supply of healthy fruits and vegetables was the most identified benefit, and in Sunsari people were found to identify multiattributes of the rooftop garden which ranks second in case of Morang. The details of the benefits of rooftop garden have been presented in Figure 6. Rooftop agriculture was recognized for resource-efficient agriculture, organic products, and intensive production of vegetables [1], which is in line with our finding, but evidence of soilless agriculture, use of GMOs, and intensive production of animal has not been reported in our survey. It is likely to have more rejection regarding the introduction of animals for commercial and household purpose in roofs.

### 4.4. Planting Material Used for Rooftop Gardening

**Figure 7:**
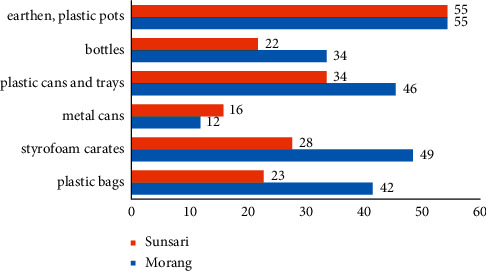
Use frequency of different planting materials in study area.

Unlike traditional farming, there are diversity of planting material being used in the study area for rooftop gardening. Earthen pots and plastic pots were used at maximum in both study areas (Figure 7). Styrofoam crate was the next abundant planting material used in Morang followed by cans and trays and metal cans being the least used. In Sunsari, plastic cans and trays are the next most used element and metal cans are the least used. Respondent's response regarding the least use of metal cans was due to the rust occurred in the cans and can cause detrimental effect on plants.

Respondents suggested to use Styrofoam crates when available as they are large, can grow deep rooted crops, and even are best for growing potatoes, but respondents have experienced the good tomato yield and continued fruiting for more than the expected time of fruiting. The main reason for the less use of Styrofoam crates in Sunsari as compared to Morang was due to unavailability and price hike in fish market due to high demand. Styrofoam carats are sold only in the fish market and have not been spotted elsewhere; most of our respondents in Sunsari and Morang told us. The use of planting material like our study has been reported by [36] in Dhulikhel, Nepal, but evidence of metal cans, earthen pots, and plastic pots were not reported which seems more economical and durable as compared to our study.

### 4.5. Determinants of Adoption of RTG

The pseudo-R2 reveals that the model fits to 37.15% and AUROC 0.8931 suggests that 89.31% of the cases are true of the predicted cases respectively. The LR Chisq. highly significant at less than 1% LOS suggests that at least one or at the most all the explanatory variables have significant effect on adoption of RTG. *R*^2^ value greater than 32 represents high impact on dependent variables [37]; i.e., all the used explanatory variables have impact on adoption of rooftop garden.

In the logit regression model, dependent variable adoption of rooftop garden was dummy (i.e., adopted = 1, not adopted = 0); the independent variables: gender, head of family, occupation, land ownership, and training were dummy; and education, family size, no. of stories, and farming year were continuous variables (Table 7). Among the tested variables, two dummy variables gender and training were found to have significant effect on adoption of rooftop garden. With the member involved in RTG management where females were high, the adoption of RTG was found to be more. If the farmer is female, then probability to adopt RTG increases by additional 30.936%.

Training was found to have significant effect on adoption of RTG for a unit increase in RTG training, and the probability of adoption was found to be increased by 11.77%. Many works of literature suggest that training has a significant effect on adoption, creating awareness in various aspects of innovation and development of agriculture [38–41]. Shrestha and Baral [42] suggest that training plays a crucial role in influencing adoption of technology which has been newly introduced. Creating awareness among farmers also has a positive role in adoption where [43] reported that awareness showed a significant effect on adoption of urban hydroponics in Kenya.

Among the tested continuous variables, schooling year, age, and, farming year are found to have significant effect on adoption of RTG. For a unit increase in schooling year of respondents, adoption of RTG increases by 2.6%. Similarly, for unit increase in age, adoption increases by 0.5% and farming experience of farmer suggests that, for 1-year increase in farming experience, the probability of adoption increases by 9.8%. Also, for a unit increase in family size, occupation, family head, land ownership, and number of stories, the adoption of RTG increases by 4.3%, 4.8%, 5.1%, 7.1%, and 0.8%, respectively. Education has a significant role on adoption of new technology. Since rooftop is old but emerging and more sustainably managed practice where in past days' roofs it was used for aesthetic purpose by use of flowers and decorative plants, nowadays it has been used for productive purposes, which helps to meet the daily dietary requirements.

### 4.6. Plant Diversity Indices

The number of species reported in the study area accounts higher for flowers followed by vegetable, fruits, and medicinal plants. The high report of flowers is due to the establishment of ornamental garden in roofs for decoration and aesthetic purposes. Rooftop garden not only houses interesting ornamentals but also is the source of nutritional supply [1, 36, 44, 45]. The diversity of plants reported in the study area has been presented in Annexures 1. Rooftop garden diversity often shares their complex and multiattribute natures as they are being adopted for nutritional, aesthetic, and environmental points of view.

The diversity profile of species classified in Table 8 shows that the overall Shannon weaver index was 3.58 and that of the flower was 3.12, the medicinal plant being the least. Shannon weaver diversity mostly ranges between 1.5 and 3.5; above 3.5 is mostly rare and is reported in the highly diverse ecosystems [20, 30]. The details of the diversity profile are shown in Table 6; however, the evenness in species of medicinal plants is higher and that of overall species is the least. The plant diversity suggests a complex nature of the farming system in roofs of Nepal as it is more common in-home and kitchen gardens. The main reason for this might be due to cultural and aesthetic values followed from past generations and adopted in rooftops too. A major difference reported in this study as compared to the home garden was ornamental plants diversity which is high in rooftop gardens, but in-home garden vegetables were more abundant and enough for home consumption [20].

### 4.7. People's Perception of Rooftop Gardens in Sustainable Farming

**Figure 8:**
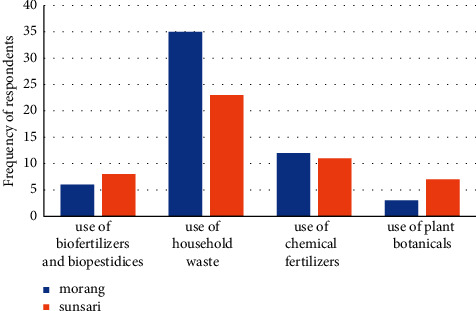
Tools of plant growth and management in rooftop gardens in study area.

Most of the rooftop garden adopters were aware of the sustainable farming as 4 respondents from Sunsari and 5 from Morang were unaware of the concept of sustainability but were aware that products obtained from rooftop are raised using an ecofriendly approach. A direct question was asked to respondents about their perception regarding sustainability of rooftop gardens and what approaches are adopted and further needed to be adopted. In the sustainable model of farming, mostly the use of chemical fertilizers and insecticides are not allowed as it disturbs the ecosystem (Figure 8). In the changing climate scenario, adoption of rooftop garden (more even in sustainable model) in developing countries is much remarkable in terms of ecological and production motive. The use of chemical pesticides has not been reported in the study for controlling insect pest in green roofs, but the use of chemical fertilizers has been reported. Declining wildlife populations in farmland and rural areas due to increased use of different chemical pesticides increased the importance of cities as wildlife refuge, while organic cultivation in rooftop farming could possibly increase plant, insect, and bird habitat in densely built environments and contribute to urban corridor networks [46]. In sustainability and influencing adoption of rooftop gardens, the multiple and significant benefits including diversity maintenance have been reported by [2]. The use of household waste, mostly biodegradable household wastes, is regarded as manure, and nonbiodegradable products like plastic bags and cans were found to be used as planting material. The use and management of household waste play an important role in management of municipal waste and local government should encourage people more to adopt green roofs. Household wastes hold a significant role in productivity and production function as it provides micro and macro nutrients and allows carbon sequestration [47]. Moreover, [12] reported that rooftop gardens provide not only production but also ecological function and carbon sequestration, along with providing a special place for optimum growth of plants promoting microorganisms [48].

### 4.8. Constraints Faced by the Respondents in RTG

**Figure 9:**
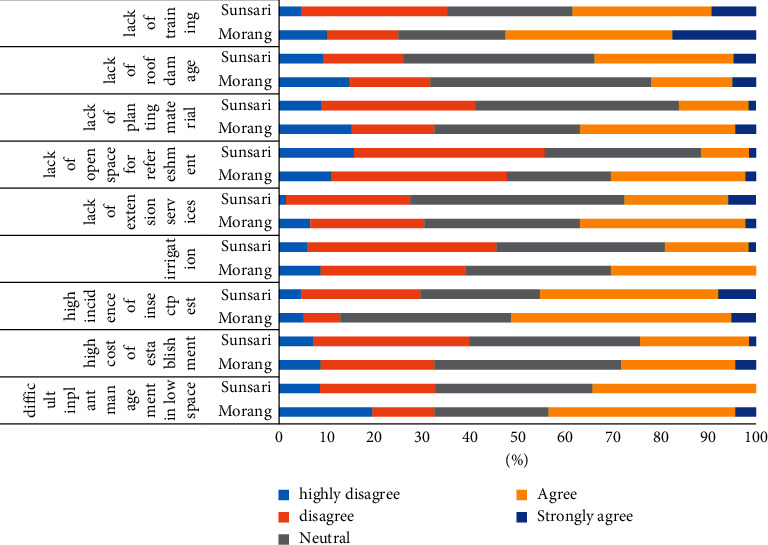
Constraints faced by rooftop adopters.

To record the constraints 5-point Likert scale technique was used and later it was converted into graph based on study area. The details of the constraints and respondent's response are as shown in Figure 9. In the Sunsari district, lack of extension service (0.73) is the most faced constraints in the RTG; it not only affects continuation but also adoption. Accordingly, difficulty in plant management (0.71), high incidence of disease and pest (0.70), lack of training (0.68), roof damage (0.67), irrigation facility (0.63), high cost of establishment (0.62), lack of planting material (0.6), and lack of open space for refreshment (0.58) are arranged based on an index of agreement.

Similarly, in Morang district also lack of extension service (0.68) was the most encountered constraint faced by the people in RTG, followed by difficulty in plant management in low space (0.5), lack of planting material (0.46), high cost of establishment (0.451), lack of training (0.45), high incidence of insect pest (0.44), irrigation (0.42), lack of open space for refreshment (0.41), and roof damage (0.39).

The higher the value of index of agreement, the higher the level of constraint and it must be prior with respect to other constraints. Lack of technical knowledge and area on roof was the major limiting factor for continuation and adoption of rooftop garden in Dhulikhel, Nepal [36]. Rooftop gardens are designed in urban setting and still naked roofs are seen due to the unavailability of leisure time, lack of manpower for management, and the fear of roof damage [4, 6].

## 5. Conclusion and Recommendation

The present study focuses on the urban households who have and have not adopted rooftop gardens, determines the factors affecting adoption of rooftop gardens, and documents the species reported in the roof of adopters. The variation in the size of roofs might also be the ruling cause of early adoption and continuation of the rooftop garden where the overall size of the roof was significantly different. Also, the study reported that the socioeconomic factor plays an important role in technology adoption and thereof in the newly emerging rooftop garden. Most adopters were highly educated, have got training related to RTG, people of younger age group, and have had farming experience. For plant growth and management use of kitchen, waste is highly used as biodegradable waste is of high nutritional importance and use of biofertilizers and promotion of organic farming have also been reported; this might be because crops are grown for household purposes. Still, constraints have been faced by adopters like lack of extension service regarding hints and tips for rooftop garden management in the study area. Thus, for a successful establishment of green roofs, local government and organizations should focus on the following:

- Provision of trainings and result demonstration to people willing to adopt RTG
- Distribution of crop calendar and package of practice to be followed and pest and disease management tactics sustainably
- Input support and training for proper use of biological fertilizers and pesticides
- Policy implication on providing grants for establishing rooftop garden so that one could easily adopt and advertise the benefits and services of rooftop gardens
- Participatory policy formulation promises and more increment of adopters

## Figures and Tables

**Table 1:** Production status and environmental benefits acquired from urban green roofs: literature review.

Crop	Year and season of research	Country of research	Objective	Major findings	Conclusion	References
22 crop species were planted (top five: tomato, chard, lettuce, pepper, and eggplant)	2015–2017	Barcelona, Spain	The objective of this research was to experiment the yield potential of the green roof under soil less polyculture	The experiment was conducted on 18 m^2^ soil with less polyculture, found productivity of 10.6 kg/m^2^/year, and reported that 5.3 m^2^ of area is required to feed one person annually	The productivity of soilless urban agriculture is high and cucurbitaceous crop had low yield compared to others. There should be cultivation-based trial on urban RTG helping to meet the market demand	[11]

Tomato and lettuce	2013–2015	AgroParis Tech university	The purpose of this study was to access the rooftops productivity using organic waste having the potential to generate many urban ecosystem services	The result is high levels of food provision with acceptable food quality in terms of contaminants, important runoff mitigation, and use of a local organic waste, but with a negative effect on runoff water quality in terms of carbon	The foods are of acceptable quality and urban organic waste has huge production potential in rooftop garden	[12]

Tomatoes (Solanum lycopersicum), beans (Phaseolus vulgaris), cucumbers (Cucumis sativus), peppers (Capsicum annuum), basil, and chives	2010-2011	United States of America	The purpose of this study was to explore three mulching strategies (pine bark, living sedum, and no mulch) and three fertilization regimens (25, 50, and 100 g·m^−2^ of 14-14-14 N-P-K slow release fertilizer applied twice each growing season) over two growing seasons to determine their benefits to rooftop agriculture	Tomato and cucumber have a positive response to fertilizer and under intensive management yield of tomato, bean and cucumber were comparable to that of conventional agriculture	More research must be conducted to access the alternative local mulching material and source of nutrients for organic production	[13]

Pepper and lettuce	June to September (2020)		To examine production systems by testing struvite solubility and uptake in granular form for two different crops: pepper plants (a highly P-demanding crop with a long growth cycle) and lettuce (shorter cycle)	The three cycles of lettuce treated with 20 g of struvite had the highest and most sustained overall yield, although such a high struvite concentration resulted in very slow dissolution. However, no signs of P deficiency can be seen in the pepper plants; even when obtaining a greater production, the P content was regarded as very low due to the slow struvite solubility	The use of struvite in hydroponic production due to the capacity of sustained production of shorter and longer cycle crops as well as the reduction of the environmental impacts compared to mineral fertilizer	[14]

Lettuce and leafy vegetables on hydroponics: tomato, chili pepper, eggplant, melon, and watermelon on soils	2012–2014	Bologna, Italy	Assessing the sensitivity of the results to the availability of reused materials and the use intensity of the community rooftop garden for producing various crops	The best techniques of lettuce cultivation to address global warming were floating in the summer, with 65–85% less environmental impact per kilogram than nutrient film, and soil production in the winter, with 85–95% less environmental impact	In the design of rooftop gardens, soil production and fruit vegetables might be prioritized to achieve higher levels of ecoefficiency. However, leafy production using the floating technique is recommended for areas where water scarcity is an environmental issue, as it is the most water-efficient option	[15]

**Table 2:** Details of the parameters used in binary logit regression model.

Parameters	Type of variable	Code used	Mean
Dependent variable			
Adoption of RTG (Y)	Dummy	1 = yes, 0 = no	0.784

Independent variable			
Gender of respondent (X1)	Dummy	(1 = male, 0 = female)	0.567
Age of respondent (X2)	Continuous	Age in years	37.77
Schooling year (X3)	Continuous	Years of schooling	12.66
Family size (X4)	Continuous	Number of members in a family	5.165
Family head (X5)	Dummy	(1 = male, 0 = female)	0.525
Occupation (X6)	Dummy	(1 = agriculture, 0 = other)	0.66
Landownership (X7)	Dummy	(1 = male, 0 = female)	0.68
Number of stories (X8)	Continuous	Number of floor in a house	3.36
Trainings (X9)	Dummy	(1 = participated, 0 = not participated)	0.59
Farming years (X10)	Continuous	Experience of farming	2.42

**Table 3:** Details of the diversity indices used for analysis.

Diversity indices	Description	Formulae	References
Shannon-weaver	It is the most popular diversity indices and rises with the rise in the several species and the evenness of their abundance. Its value ranges from 0 to 5 and typically from 1.5 to 3.5.	*H*=∑_*i*=1_^*n*^*pi∗* ln(*pi*)*Pi* = fraction of the sampled population made of species *i*; *n* = number of species reported; ∑ = sum from species 1 to species and “ln” is the natural logarithm to the base e (log).	[20, 31–33]

Simpson 1-D	D measures the dominance and 1-D measures the species diversity estimate. Its value ranges from 0 to 1	*D* = Ʃ(*n*/*N*)^2^, where *N* = total number of all species; *n* = total number of a particular species	[32, 34]

Evenness measure	Evenness is measured as *E* = *H*/*H*_max_, i.e., the ratio of observed diversity to maximum diversity [34]		

The effective number of species	The exponential value of the Shannon–Weaver diversity index results in the effective number of species as explained by Peet [32]		

**Table 4:** Socioeconomic characteristics of the study area.

Parameter	Morang	Sunsari	Total
Gender			
Male	33	30	63
Female	25	28	53
Household head			
Male	31	39	70
Female	19	11	30
Both	8	8	16
Decision making			
Male	29	33	62
Female	17	11	28
Both	12	14	26
Age	38.47 ± 0.205	42.14 ± 0.725	37.77 ± 0.43
Caste			
Chhetri	15	17	32
Brahmin	25	13	38
Madhesis	11	7	18
Janajati	2	24	26
Dalits	0	2	2
Occupation			
Agriculture	17	20	37
Other	41	38	79
Family size	5.14 ± 0.197	5.19 ± 0.214	5.165 ± 0.146
Dependency ratio	2.12 ± 0.118	2.14 ± 0.146	2.13 ± 0.112
Schooling year	11.28 ± 0.652	11.48 ± 0.609	12.66 ± 0.613

**Table 5:** Status of roof and garden as per adopters vs. nonadopters.

Parameter	Adopters	Nonadopters	*F*-value	*P*-value
Size of roof in (m^2^)	181.98	142.40	0.65^*∗*^	0.05
(%) Covered by rooftop garden	34.09	28.29	0.989^ns^	0.645

**Table 6:** Status of roof and garden based on study area.

Parameter	Morang	Sunsari	*F*-value	*P*-value
Size of roof in (m^2^)	150.69	174.421	1.39^ns^	0.105
Area of roof under farming	13.46 (3–140)	24.17 (7–150)	2.19	≤0.0001
(%) Covered by rooftop garden	30.5	33.20	1.68^*∗*^	0.0248

**Table 7:** Factors affecting in adoption of rooftop garden: an analysis using binary logit model.

Have roof top farm	Coef.	Std.	*z*	*P* > *z*	dy/dx
Gender	−2.96052	0.827985	−3.58	≤0.0001	−0.30936^*∗∗*^
Age	0.057204	0.028724	1.99	0.046	0.005978^*∗*^
Schooling year	0.254438	0.11518	2.21	0.027	0.026588^*∗*^
Family size	0.413564	0.307696	1.34	0.179	0.043216
Family head	0.488109	0.643735	0.76	0.448	0.051006
Occupation	0.46092	0.68697	0.67	0.502	0.048164
Landownership	0.686592	0.710618	0.97	0.334	0.071746
Number of stories	0.078205	0.317562	0.25	0.805	0.008172
Trainings	1.12708	0.616496	1.83	0.05	0.117776^*∗*^
Farming years	0.94502	0.303067	3.12	0.002	0.098751^*∗*^
Cons	−7.18738	3.191387	−2.25	0.024	

Number of obs = 116, LR chi2 = 44.92, Prob > chi2 ≤  0.0001, Pseudo-*R*^2^ = 0.3715, Log likelihood = -37.997031. ^*∗*^: significant at 5% LOS, and ^*∗∗*^: significant at 1% LOS.

**Table 8:** Diversity indices of species reported in the study area.

Criteria	Shannon (H)	Simpson 1-D	Evenness	Effective number of species
Overall	3.58	0.95	0.38	35.87
Vegetable	2.08	0.84	0.4	8
Fruit	1.89	0.79	0.57	6.6
Flower	3.12	0.94	0.51	22.64
Medicine	1.67	0.78	0.67	5.3

## Data Availability

The data can be obtained from the corresponding author upon request.

## Supplementary Materials

Supplementary Materials Annex 1. Diversity of crops reported from the study area.
