# Supplementary material for: Assessment of the Status of Rooftop Garden, Its Diversity, and Determinants of Urban Green Roofs in Nepal
Source: Scientifica (Cairo). 2022 Jun 6;2022:6744042. doi: 10.1155/2022/6744042 (PMC9192269; doi:10.1155/2022/6744042)
Supplement: Supplementary Materials — Annex 1. Diversity of crops reported from the study area. [file 6744042.f1.docx]

**Annexures**

Annex 1. Diversity of crops reported from Study area.

| Crops | Family | *Scientific Name* |
| --- | --- | --- |
| **Vegetables** |  |  |
| Tomato | Solanaceae | *Solanum lycopersicum* [*L.*](http://www.theplantlist.org/tpl1.1/record/tro-29605838) |
| Cauliflower | Brassicaceae | *Brassica oleracea**L.* |
| Potato | Solanaceae | *Solanum**tuberosum**L.* |
| Taro | Araceae | *Colocasiaesculenta**(L.) Schott* |
| Coriander | Apiaceae | *Coriandrum**sativum**L.* |
| Pumpkin | Cucurbitaceae | *Cucurbita pepo L.* |
| Brinjal | Solanaceae | *Solanum**melongenaL.* |
| Garlic | Amaryllidaceae | *Allium sativum**L.* |
| Onion | Amaryllidaceae | *Allium**cepaL.* |
| Chilly | Solanaceae | *Capsicum frutescens L.* |
| Bitter Gourd | Cucurbitaceae | *Momordica**charantiaL.* |
| Bottle Gourd | Cucurbitaceae | *Lagenariasiceraria(Molina) Standl.* |

| Mustard | Cruciferae | *Brassica**juncea(L.) Czern.* |
| --- | --- | --- |
| Cucumber | Cucurbitaceae | *Cucumis**sativus**L.* |
| Sweet chart | Convolvulaceae | *Ipomoea**batatas**(L.) Lam.* |
| Japanese Melon | Cucurbitaceae | *Cucumis melo L.* |
| Asparagus | Asparagaceae | *Asparagus**officinalis**L.* |
| Chayote | Cucurbitaceae | *Sechiumedule(Jacq.) Sw.* |
| Garden cress | Brassicaceae | *Lepidium**sativum**L.* |
| Cabbage | Brassicaceae | *Brassica oleraceae var. capitata* |
| **Fruits** |  |  |
| Pomegranate | Punicaceae | *Punica granatum L.* |
| Guava | Myrtaceae | *Psidium**guajavaL.* |
| Balsam Apple | Cucurbitaceae | *Momordica balsamina L.* |
| Lime | Rutaceae | *Citrus lemon L.* |
| Lemon | Rutaceae | *Citrus sp.* |
| Dragon Fruit | Cactacea | *Hylocereus undatus (Haw.) Britton & Rose* |
|  |  |  |
| **Medicinal plant** |  |  |
| Dubo | Poaceae | *Cynodondactylon(L.) Pers.* |
| Mint | Menthaceae | *Mentha sp.* |
| Tulasi | Menthaceae | *OcimumtenuiflorumL.* |
| Saunf | Apiaceae | *Foeniculumvulgare**Mill.* |
| Lemon grass | Poacea | *Cymbopogon citratus (DC.) Stapf* |
| Dhupi | Cupressaceae | *Cryptomeria japonica D. Don* |
| Ganja | Cannabaceae. | *Cannabis sativa* |
| Bay leaves | Lauraceae | *Laurus nobilis L.* |
| cinnamon | Lauraceae | *Cinnamomum verum* |
| **Flowers** |  |  |
| Hibiscus | Malvaceae | *Hibiscus**rosa*[*-sinensis*](http://www.theplantlist.org/tpl1.1/record/kew-2850448) *L.* |
| Money Plant | Araceae |  |
| Aleovera | Asphodelaceae | *Aloe vera* |
| Marigold | Asteraceae | *Tagetes patula L.* |
| Bryophyllum | Crassulaceae | *Bryophyllum pinnatum* |
| Senecio | Asteraceae | *Senecio radicans* |
| Periwinkle | Apocynaceae | *Catharanthusroseus**(L.) G.Don* |
| Rose | Rosaceae | *Rosa sp.* |
| Chrysenthemun | Asteraceae | *Chrysanthemum indicum L.* |
| Dahlia | Asteraceae | *Dahlia sp.* |
| Crown of thorns | Euphorbiaciae | *Euphorbia**miliiDes Moul.* |
| Honeysuckle | Loniceraceae | *Loincera sp.* |
| Snapdragon | Plantaginaceae | *Antirrhinum majus* |
| Dianthus | Caryophyllaceae | *Dianthus**caryophyllusL.* |
| Garden Geranium | Geraniaceae | *Pelargonium × hortorum L.H. Bailey* |
| Jasmine | Jasminaceae | *Jasminium polyanthum* |
| Schefflera | Araliaceae | *Schefflera arboricola (Hayata) Merr.* |
| Sage | Lamiaceae | *Salvia officinalis* |
| Bougainvillea | Nyctaginaceae | *Bougainvillea glabra* |
| Creeping Woodsorrel | Oxalidaceae | *Oxalis corniculata* |
| Poppy | Papaveraceae | *Papaver somniferum. L.* |
| Umbrella Tree | Araliaceae | *Schefflera actinophylla (Endl.) Harms* |
| Surb Medium size | - | *-* |
| Rani salla | Pinaceae | *Pinus roxburgii* |
| 9 o’clock flower | Portulacaceae | *Portulaca grandiflora* |
| Burgenda Surb | Calycanthaceae | *--* |
| Buttercup | Ranunculaceae | *Ranunculus asiaticus* |
| Helinium | Heleniaceae | *Helinium autumnale* |
| Decorative fern | Asparagaceae | *--* |
| Hollyhock | Malvaceae | *Alcea rosea* |
| Acorus | Acoraceae | *Acorus calamus* |
| Daffodil | Amaryllidaceae | *Narcissus sp.* |
| Cactus | Cactaceae | *Cactus sp.* |
| Basil | Lamiaceae | *OcimumbasilicumL.* |
| Poinsettia | Euphorbiaceae | *Euphorbia**pulcherrimaWilld. ex Klotzsch* |
| Saliva | Apodidae | *Salvia officinalis* |
| Daisy | Asteraceae | *Bellis perennis**L.* |
